# Psychometric Evaluation of Anxiety, Depression, and Sleep Quality after a Mild Traumatic Brain Injury: A Longitudinal Study

**DOI:** 10.1155/2019/4364592

**Published:** 2019-04-14

**Authors:** Hon-Ping Ma, Po-Shen Chen, Chung-Shun Wong, Cheng-Fu Chang, Ju-Chi Ou, Yan-Rou Tsai, Wen-Ta Chiu, Shin-Han Tsai, Kuo-Hsing Liao, Yung-Hsiao Chiang, Jia-Yi Wang, Kai-Yun Chen, John Chung-Che Wu

**Affiliations:** ^1^Department of Emergency Medicine, Shuang Ho Hospital, Taipei Medical University, New Taipei City, Taiwan; ^2^Department of Emergency Medicine, School of Medicine, Taipei Medical University, Taipei, Taiwan; ^3^Graduate Institute of Injury Prevention and Control, Taipei Medical University, Taipei, Taiwan; ^4^Department of Neurosurgery, Taipei City Hospital, Zhongxiao Branch, Taiwan; ^5^Department of Surgery, College of Medicine, Taipei Medical University, Taipei, Taiwan; ^6^Graduate Institute of Neural Regenerative Medicine, College of Medical Science and Technology, Taipei, Taiwan; ^7^Center for Neurotrauma and Neuroregeneration, Taipei Medical University, Taipei, Taiwan; ^8^Institute of Injury Prevention and Control, College of Public Health and Nutrition, Taipei Medical University, Taipei, Taiwan; ^9^Department of Neurosurgery, Wan Fang Hospital, Taipei Medical University, Taipei, Taiwan; ^10^Department of Neurosurgery, Taipei Medical University Hospital, Taipei, Taiwan; ^11^Graduate Institute of Medical Sciences, College of Medicine, Taipei Medical University, Taipei, Taiwan

## Abstract

*Introduction*. Over 1 million mild traumatic brain injury (mTBI) cases are reported annually worldwide and may result in cognitive, physical, and emotional deterioration; depression; anxiety; and sleep problems. However, studies on long-term mTBI effects are limited. This study included 440 patients, and regular follow-ups of psychological assessments were performed for 2 years. Four questionnaires, including the Pittsburgh sleep quality index (PSQI), Epworth sleepiness scale (ESS), Beck's anxiety inventory (BAI), and Beck's depression inventory (BDI), were used to evaluate sleep problems, daytime sleepiness, anxiety, and depression, respectively. Results show that BAI and BDI scores considerably improved at the 6th-week, 1st-year, and 2nd-year follow-ups compared to baseline, yet these remained significantly different. In addition, anxiety and depression were prominent symptoms in a select subgroup of patients with poor initial evaluations, which improved over the 2 years. However, the ESS and PSQI scores fluctuated only mildly over the same time span. In conclusion, the mTBI patients showed a gradual improvement of anxiety and depression over the 2 years following injury. While anxiety and depression levels for mTBI patients in general did not return to premorbid status, improvements were observed. Sleep disorders persisted and were consistent with initial levels of distress.

## 1. Introduction

Traumatic brain injury (TBI) is a major public health issue listed in the World Health Organization, and it can lead to acute and chronic long-term neuropsychiatric and cognition dysfunction [1–3]. More than 80% of these injuries were classified as mild [4], named mild traumatic brain injury (mTBI). An estimated 1.7 million people per year sustain a TBI, and cumulatively over 5.3 million people in the USA have a TBI-induced disability [5, 6]. Two population-based studies using the Taiwan National Health Insurance Database indicated that TBI was associated with a 1.68 times greater risk of dementia, and the pathogenic hazard ratio for repeated TBI patients further increased to a 3.62 times greater risk of developing dementia [7, 8]. mTBI is considered a significant risk factor for neurodegenerative diseases, including Parkinson's disease, Alzheimer's disease, and dementia [9–11]. Approximately 70-90% of mTBI patients continue to experience neurocognitive dysfunction, although several patients could resolve such problems within the first year post injury [12, 13].

TBI could result in cognitive, social, emotional, physical, and behavioral symptoms, such as headaches, sleep disturbance, depression/anxiety, and dizziness [14–21]. Most patients with mTBI recovered within 6 months post injury, but some do not. The average healthcare costs of the mTBI group were 76% higher than those without mTBI in the 3 years after injury. In addition, the costs of treating psychiatric illnesses were more than double the total cost for nonpsychiatric patients [22].

The symptoms and related trials of mTBI were studied primarily in military service members and veterans for blast-related mTBI [23–25]. However, there have been no studies on the long-term clinical effects of mTBI with blast-free etiologies. The purpose of this study is to analyze the psychological symptoms caused by mTBI in a prospective group of subjects.

## 2. Methods

### 2.1. Participants

**Figure 1:**
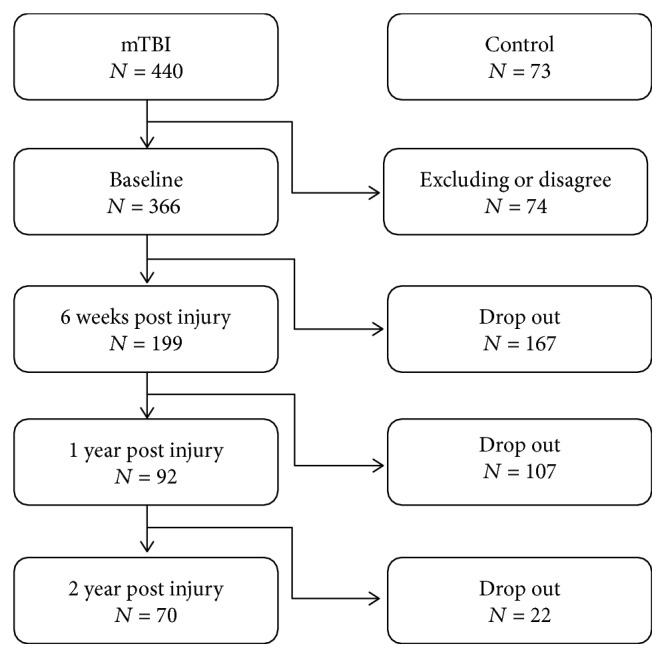
Flow diagram of study recruitment.

Between September 2010 and October 2016, participants with mTBI were recruited from admissions to three hospitals in Taipei: Taipei Medical University Hospital, Wan Fang Hospital, and Shuang Ho Hospital. The mTBI were defined according to the US Centers for Disease Control and Prevention [26] and the World Health Organization Task Force on mTBI [27]. The inclusion criteria were (1) injury to the head, (2) loss of consciousness for no longer than 30 minutes post injury, (3) Glasgow coma scale scores 13-15, (4) age between 20 and 70 years, (5) negative finding on computed tomography scans, and (6) no previous history of head injury. The exclusion criteria were (1) history of moderate or severe TBI, (2) history of mental illness, (3) history of epilepsy, and (4) pregnancy. A total of 440 patients with mTBI were screened, and 366 patients who met preliminary eligibility criteria were recruited and were asked to sign informed consent forms. The Taipei Medical University-Joint Institutional Review Board (TMU-JIRB) approved the study. Around 81% (296 of 366) of the eligible participants did not complete the 2-year follow-up (see Figure 1 for study flow). Eventually, only 70 participants were included in this longitudinal study. The control group comprised healthy participants also aged 20 to 70 years. Volunteers were recruited through community advertising and referrals and the only inclusion criterion was a lack of previous head injury.

### 2.2. Outcome Measures

#### 2.2.1. Beck's Anxiety Inventory (BAI)/Beck's Depression Inventory (BDI)

Anxiety symptoms were evaluated by the BAI [28], which includes 21 items scored from 0 to 3 to generate a total score ranging from 0 (no problem) to 63 (severe problem). The severity of depression was assessed via the BDI-II [29], which is comprised of 21 items scored from 0 to 3 to generate a total score ranging from 0 to 63. Higher scores indicate clinically significant anxiety/depression.

#### 2.2.2. Epworth Sleepiness Scale (ESS)

The severity of daytime dozing was assessed via ESS [30]. The questionnaire contained 8 items scored on a 4-point scale; score 0 (never doze), score 1 (slight chance), score 2 (moderate chance), and score 3 (high chance of dozing), to generate a total score ranging from 0 (no problem) to 24 (severe problem). The clinical cut point for ESS is 10 and an ESS score > 10 indicated excessive daytime sleepiness.

#### 2.2.3. Pittsburgh Sleep Quality Index (PSQI)

Sleep quality and sleep dysfunction were evaluated by a self-reported questionnaire, PSQI [31]. It consists of 19 questions to measure 7 components: duration of sleep, sleep disturbance, sleep latency, sleep dysfunction due to sleepiness, sleep efficiency, overall sleep quality, and requirement of medication to sleep. Each component is scored from 0 to 3 generating a total score ranging from 0 to 21. A total score of greater than 5 is indicative of clinically significant poor sleep quality.

From a diagnostic perspective, the clinical end points for the four outcomes were set previously. A BAI score > 7 and a BDI score > 9 are indicative of meaningful anxiety and depression. Similarly, an ESS score > 10 and a PSQI score > 5 indicated substantial daytime sleepiness and sleep disturbance. The subgroups' improvements, according to the clinical cut points, were analyzed for 2 years post injury.

### 2.3. Statistical Method

The demographic variables were compared via the Student *t*-test and Mann–Whitney *U* test for normally distributed and abnormally distributed continuous variables, respectively. The categorical variables were compared between the two groups via the chi-squared test (gender and mechanism of injury). Outcome scores were compared to the general control group scores to reveal any improvements. Additionally, a generalized linear mixed model approach was performed to analyze the participants' pattern of scores in anxiety, depression, daytime sleepiness, and sleep disturbance across the 2 years post injury for longitudinal investigation. The significance level was set at 0.05 for all analyses. The statistical software R version 3.4.0 (copyright (©) 2017, The R Foundation for Statistical Computing) was employed to analyze the longitudinal data.

## 3. Results

70 (19.12%) of the original 366 mTBI participants completed the 2-year follow-up study, and 296 patients withdrew. The demographic information of this study is shown in Table 1. The average ages for both groups were 41.37 and 31.10 years, respectively. There were 38 (54.29%) and 182 (61.49%) women in both groups with an average GCS of 14.93 and 14.94, respectively. About one-third of the participants had mTBI caused by falls, and about half had injuries caused by traffic accidents. The average outcome scores of the participants who completed the 2-year assessment were 9.30, 9.37, 7.37, and 8.26 for anxiety, depression, daytime sleepiness, and sleep quality, respectively. The patients who dropped out from this study had an average anxiety score of 8.81, depression score of 9.01, daytime sleepiness score of 7.54, and sleep quality score of 7.73. Overall, there was no significant difference between the participants who completed or dropped out from this study, suggesting that these participants dropped out at random.

In order to delineate the differences between participants with and without head injuries, healthy control participants were recruited and their results are shown in Table 2. Between the mTBI and control groups, there were no differences in average age, average GCS, gender, average final education year, and percentage of smokers. All questionnaire scores were significantly different between the control group and the mTBI group, with the exception of the daytime sleepiness scores. The scores of anxiety, depression, and sleep quality of the mTBI group were considerably higher than those of the control group, suggesting that participants who had suffered a head injury exhibited symptoms of anxiety, depression, and sleep quality.

There were four time points for assessment for the mTBI group: initial (baseline), 6th week, 1st year, and 2nd year; there was one assessment for the control group. In order to measure improvement, the scores between the mTBI and control groups were compared, and the results are summarized in Table 3. In the mTBI group, the average anxiety score improved from 9.30 at the initial assessment to 4.70 at the 2-year postinjury assessment. However, the average score for anxiety was 2.27 (standard deviation 3.62) in the healthy control group. Thus, the average score for anxiety in the mTBI group remained significantly different from that of the control group after 2 years (*p* = <0.01). The average depression score was 9.37 at the initial assessment and decreased to 5.49 at the 2-year assessment for the mTBI group. In the control group, the average depression score was 3.18 (±4.53), and this was significantly different from that in the mTBI group at all four assessments.

In addition, the average sleep quality score of the mTBI group at the initial assessment was high at 8.26, and this was significantly different from that of the control group at 5.84 with a standard deviation of 2.32 (*p* = <0.01). Over the course of 2 years, the average sleep quality score of the TBI group remained steady at around 7 or 8, and this was significantly different from that of the control group (*p* = <0.01). However, there was no significant difference in the average daytime sleepiness scores between the control and the mTBI groups at the initial assessment (5.97 and 7.37, respectively, *p* = 0.16). At the 2-year assessment, the mTBI group score had a negligible increase to 6.97, yet the difference to the control group remained insignificant (*p* = 0.28).

Furthermore, the sleep quality score of the mTBI group at the initial assessment was high at 8.26, and this was significantly different from that of the control group at 5.84, with a standard deviation of 2.32. Over the course of two years, the average scores of sleep quality were around 7 and 8, and these continued to be statistically significantly different from that of the control group. Again, there was no difference between the control and the mTBI groups at the initial assessment with respect to the daytime sleepiness scores (average 5.97 and 7.37, respectively). Over the 2-year period, there was no difference in the scores of daytime sleepiness between the control and mTBI groups.

**Figure 2:**
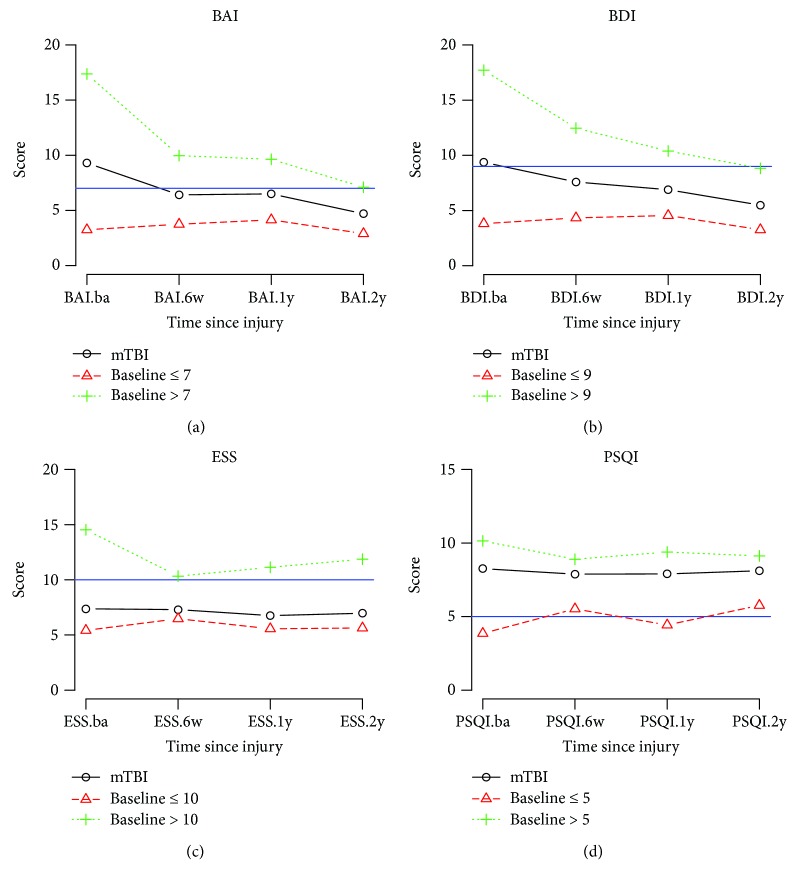
Average group scores across the four symptom categories. (a) Mean BAI (Beck's anxiety inventory) scores of two groups (BAI > 7 (+) versus BAI ≤ 7 (∆)) across four assessments at baseline, 6th week, 1st year, and 2nd year. (b) Mean BDI (Beck's depression inventory) scores of two groups (BDI > 9 (+) versus BDI ≤ 9 (∆)) across four assessments. (c) Mean ESS (Epworth sleepiness scale) scores of two groups (ESS > 10 (+) versus ESS ≤ 10 (∆)) across four assessments. (d) Mean PSQI (Pittsburgh sleep quality index) scores of two groups (PSQI > 5 (+) versus PSQI ≤ 5 (∆)) across four assessments.

The mTBI participants were divided into two subgroups, good and poor. The comparison results are shown in Table 4, and the 2-year trends are illustrated in Figure 2.

### 3.1. Anxiety

30 participants reported symptoms of anxiety at the initial assessment, with an average score of 17.37. The average score for the anxiety (poor) group was significantly different from the average score for the anxiety-free (good) group (*p* = <0.01). In the anxiety group, the score improved with time. However, the average score at the 2nd year of assessment (7.10) remained over the clinical end point. Therefore, the majority of the anxiety group continued to experience symptoms of anxiety at the 2nd year of assessment. The anxiety-free participants remained as such at the 2-year assessment.

### 3.2. Depression

At the initial assessment, 28 mTBI participants reported depression with an average score of 16.96 (±10.02), and 42 mTBI participants were in the depression-free group with an average score of 4.19 (±3.11). The average depression scores were significantly different between the depression (poor) and depression-free (good) groups at all assessments (*p* = <0.01). At the 2-year postinjury assessment, the average score of 8.82 in the depression group was lower than the clinical end point (9). That is, the reported symptoms of depression improved after 2 years. However, the average score remained higher than that of the depression-free TBI group (3.26).

### 3.3. Daytime Sleepiness

Most participants (55, 78.57%) were free of daytime sleepiness; however, several participants (15, 21.43%) reported that they experienced these symptoms. The daytime sleepiness (poor) group had an average score of 10.33 at the 6th week which was above the clinical end point (10), and participants in the problem-free (good) group had average ESS scores around 5-6 across the four assessment points.

### 3.4. Sleep Quality

The majority of the mTBI participants (49, 70%) reported poor sleep quality, and 21 participants (30%) reported good sleep quality at the initial assessment. At the 6-week assessment, there were reports of sleep problems in the good sleep quality group and improvements in the poor sleep quality group. However, the average scores of these groups did not significantly change over the 2-year course. In addition, the differences of average PSQI scores between the two groups were significant at each assessment.

**Figure 3:**
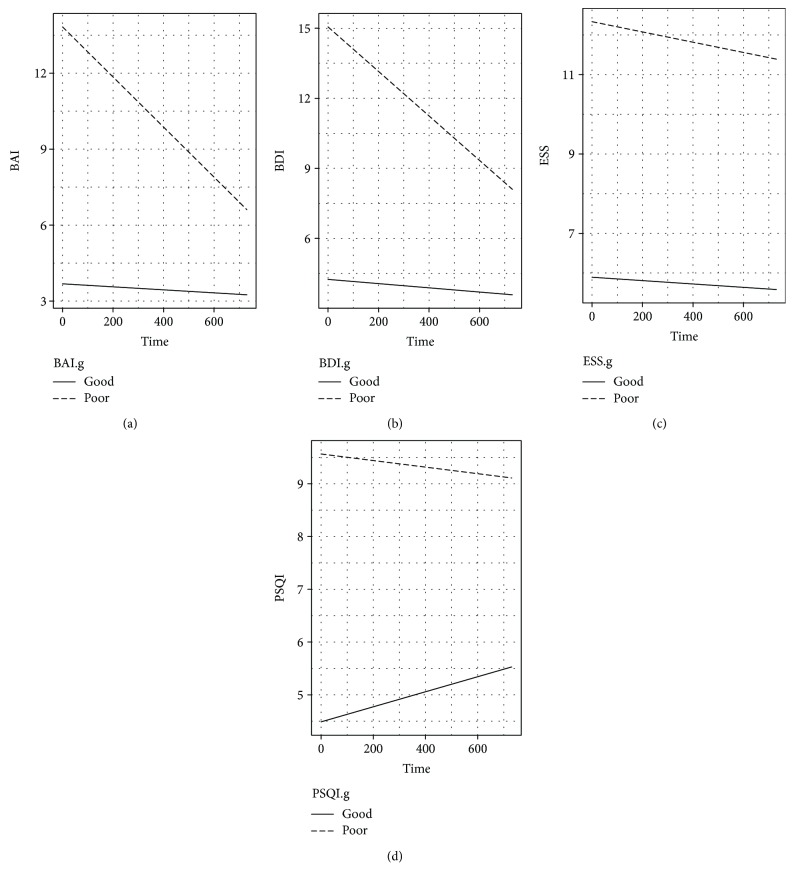
Estimated trends for four outcomes between the good and poor groups in the mTBI patients. (a) BAI (Beck's anxiety inventory) score ≤ 7 (good: solid line) versus score > 7 (poor: dashed line). (b) BDI (Beck's depression inventory) score ≤ 9 (good: solid line) versus score > 9 (poor: dashed line). (c) ESS (Epworth sleepiness scale) score ≤ 10 (good: solid line) versus score > 10 (poor: dashed line). (d) PSQI (Pittsburgh sleep quality index) score ≤ 5 (good: solid line) versus score > 5 (poor: dashed line).

For each individual, the changes in all outcomes were evaluated using an individual growth model. The physical health change by group (good-poor) is demonstrated in Figure 3, and the individual growth models for longitudinal changes in anxiety, depression, and sleep quality are described in Table 5. The intercept for scores of the daytime sleepiness and sleep quality was significant, indicating that individual scores varied for daytime sleepiness and sleep quality. The variable, age, was statistically significant in the depression category only. The group effects were significant for all physical health outcomes, indicating that the longitudinal changes in anxiety, depression, daytime sleepiness, and sleep quality after mTBI were dependent on their initial status (BAI and BDI scores at baseline). In addition, the interaction effects between the groups and postinjury time were significant in the anxiety and depression categories. That is, the longitudinal changes in anxiety and depression were dependent on postinjury time and good-poor groups.

## 4. Discussion

This study investigated the effects of mTBI over the course of 2 years. To our knowledge, it is the first longitudinal study that has focused on the anxiety, depression, and sleep quality outcomes of patients following mTBI.

In this study, epidemiological comparisons between the mTBI and control groups were largely similar. The two groups did not exhibit statistically significant differences with respect to age, gender, GCS upon arrival, education, and smoking. However, we observed a significantly higher percentage of subjects who consumed alcohol in the control group compared to mTBI patients (57.5% vs. 42.8%). Despite this epidemiological difference, our results yielded significant findings that allow for a better understanding of mTBI patients.

The investigation into the dropouts also provided a better understanding for the study. Despite a total number of 440 patients initially enrolled in the study and 366 patients who completed the baseline evaluations, 296 patients withdrew, and only 70 patients were able to complete all four assessments. The 80.8% dropout rate was significant, and it required investigation. Fortunately, based on the initial baseline evaluations, the age, gender, GCS, and injury mechanisms of the subjects who dropped out were not significantly different compared to those who completed the study. This suggests that the patients who completed the evaluations at all four time points could be representative of the patients who dropped out of the study.

The results of BAI, BDI, and PSQI were significantly different in the mTBI group compared to the controls. Not only were these parameters increased following mTBI (Table 3), they also showed significant improvement at the 6th week, 1st year, and 2nd year, after baseline values were established (Table 4). While the mTBI ESS score was not significantly different compared to the control groups, there was a significant difference when compared to baseline values. The significant differences in BAI, BDI, and PSQI scores compared to the control group suggested continuous alteration in anxiety, depression, and sleep quality symptoms following mTBI, even at 2 years post injury. Additionally, subsequent to age adjustment, all parameters were significant when compared to the control group (Table 5). Fortunately, anxiety and depression symptoms (BAI and BDI) continued to improve after 2 years (Figure 2), even in cases of incomplete recovery. Previous studies demonstrated an improvement of sleep at the 6th week after injury [21], and our study confirmed such findings at the 6th week. The sleep quality symptoms (ESS and PSQI), however, worsened at later follow-ups, and appeared significantly worse than in the control group and the baseline values. In other words, while improvement in sleep is clear from baseline to the 2-year follow-up, the deterioration of ESS and PSQI from the 6-week follow-up to the 2-year follow-up is also evident. This delay in the deterioration of sleep quality deserves further attention and additional studies.

Individual end points were selected for each of the BAI, BDI, ESS, and PSQI scores, and the results revealed differences in those above and below the end points. The results were categorized into 2 subgroups for each outcome, and significantly different recovery curves were observed. The BAI and BDI groups yielded a continually improving curve for mTBI patients scoring above the end point. It was found that these scores improved to, but did not surpass, the end point. For ESS and PSQI, the groups with baseline scores above the end point continued to score above the end, and little improvement was observed in the 2 years following mTBI. Notably, those with lower PSQI scores worsened over 2 years (Figure 3), an interesting finding that would require a larger study to confirm.

This study is somewhat limited by the number of subjects. Despite the initial enrollment of 440 patients, only 70 participants completed the 6th-week, 1st-year, and 2nd-year follow-ups. The 80.8% dropout rate was unfortunate and high; however, this is not uncommon for mTBI studies. For example, a study conducted by Warren et al. witnessed a 53% dropout rate in the 6-month follow-up [32]. The high dropout rate here may pose a bias for the outcomes of the study, despite a statistically insignificant comparison between the dropouts and those who completed the study. We hope to continue the study and analysis of mTBI patients to confirm the results of this investigation in the near future.

Understanding the prevalence of anxiety and depression in mTBI patients is a first step to understanding the issues faced during treatment. Additional efforts in the treatment of these symptoms, and modulating the mechanisms that lead to them, requires further research.

## 5. Conclusion

This study shed light on mTBI with the subsequent presentation of anxiety and depression sustaining a time-dependent improvement over 2 years. The unresolved issues about the delay in the deterioration of sleep quality, paradoxical PSQI score, and the role of the modulating mechanism deserve further study despite the limited patient number and the current small scale and limited enrollment rate.

## Figures and Tables

**Table 1:** The baseline scores and characteristics of patients who followed and dropped out, at 2 years post injury.

	Followed (*n* = 70)	Dropped (*n* = 296)	*P* value
Age	41.37	39.10	0.16
Gender (F/M)	38/32	182/114	0.26
GCS	14.93	14.94	0.78
*Injury mechanism*			0.91
Falls	20 (28.57%)	91 (30.74%)	
Traffic accident	36 (51.42%)	144 (48.65%)	
Others	14 (20%)	61 (20.61%)	
*Questionnaires*			
BAI	9.30 ± 9.21	8.81 ± 9.49	0.39
BDI	9.37 ± 8.98	9.01 ± 8.46	0.76
ESS	7.37 ± 4.67	7.54 ± 4.38	0.55
PSQI	8.26 ± 4.04	7.73 ± 4.18	0.29

**Table 2:** Demographic data for the mTBI and control groups.

	mTBI (*n* = 70)	Control (*n* = 73)	*P* value
Age	41.37 ± 13.70	45.99 ± 16.27	0.08
Gender (F/M)	38/32	39/34	0.97
GCS	14.93	15.00	0.99
Education (year)	14.06	13.90	0.61
Drink (Y/N)	30/40	42/31	0.02^∗^
Smoke (Y/N)	17/53	12/61	0.11
*Injury mechanism*			
Falls	20 (28.57%)		
Traffic accident	36 (51.43%)		
Others	14 (20%)		
*Questionnaires*			
BAI	9.30 ± 9.21	2.27 ± 3.62	<0.01^∗^
BDI	9.37 ± 8.98	3.18 ± 4.53	<0.01^∗^
ESS	7.37 ± 4.67	5.97 ± 3.48	0.16
PSQI	8.26 ± 4.04	5.84 ± 2.32	<0.01^∗^

^∗^Difference is significant at 0.05.

**Table 3:** Difference between the control group and the mTBI group at each assessment (mean, standard deviation).

Mean ± SD (*P* value)	Case number	BAI	BDI	ESS	PSQI
Control	73	2.27 ± 3.62	3.18 ± 4.53	5.97 ± 3.48	5.84 ± 2.32
mTBI-baseline *P* value	70	9.30 ± 9.21 (*<0.01*)	9.37 ± 8.98 (*<0.01*)	7.37 ± 4.67 (0.16)	8.26 ± 4.04 (*<0.01*)
mTBI-6th week *P* value		6.41 ± 7.21 (*<0.01*)	7.59 ± 7.16 (*<0.01*)	7.30 ± 4.05 (0.06)	7.89 ± 3.34 (*<0.01*)
mTBI-1st year *P* value		6.50 ± 8.93 (*<0.01)*	6.89 ± 7.85 (*<0.01*)	6.76 ± 4.32 (0.42)	7.90 ± 4.33 (*<0.01*)
mTBI-2nd year *P* value		4.70 ± 6.69 (*<0.01*)	5.49 ± 6.64 (*0.02*)	6.97 ± 4.41 (0.28)	8.11 ± 3.86 (*<0.01*)

**Table 4:** Differences between the poor and good groups across the four outcomes (mean ± SD).

Outcome	Time of evaluation	Poor (*n* = 30)	Good (*n* = 40)	*P* value
BAI	Baseline	17.37 ± 8.85	3.25 ± 2.05	<0.01^∗^
	6 weeks	9.97 ± 9.21	3.75 ± 3.43	<0.01^∗^
	1 year	9.63 ± 10.72	4.15 ± 6.51	<0.01^∗^
	2 years	7.10 ± 8.79	2.90 ± 3.76	0.03^∗^

Outcome	Time of evaluation	Poor (*n* = 28)	Good (*n* = 42)	*P* value
BDI	Baseline	16.96 ± 10.02	4.19 ± 3.11	<0.01^∗^
	6 weeks	12.46 ± 8.34	4.33 ± 3.67	<0.01^∗^
	1 year	10.39 ± 9.23	4.55 ± 5.79	<0.01^∗^
	2 years	8.82 ± 8.41	3.26 ± 3.86	<0.01^∗^

Outcome	Time of evaluation	Poor (*n* = 15)	Good (*n* = 55)	*P* value
ESS	Baseline	14.53 ± 2.67	5.42 ± 2.81	<0.01^∗^
	6 weeks	10.33 ± 3.13	6.47 ± 3.89	<0.01^∗^
	1 year	11.13 ± 2.77	5.56 ± 3.88	<0.01^∗^
	2 years	11.87 ± 4.44	5.64 ± 3.35	<0.01^∗^

Outcome	Time of evaluation	Poor (*n* = 49)	Good (*n* = 21)	*P* value
PSQI	Baseline	10.14 ± 3.30	3.86 ± 1.11	<0.01^∗^
	6 weeks	8.90 ± 2.85	5.52 ± 3.25	<0.01^∗^
	1 year	9.39 ± 4.17	4.43 ± 2.18	<0.01^∗^
	2 years	9.12 ± 3.63	5.76 ± 3.39	<0.01^∗^

^∗^Difference is significant at 0.05. Time: baseline = 0, 6 weeks = 42 days, 1 year = 365 days, and 2 years = 730 days.

**Table 5:** Parameter estimates from the growth model for the four outcomes with random group effect.

Outcome	Variables	Estimate	*t*	*P* value
BAI	Intercept	0.513	0.241	0.810
	Time	−0.001	−0.419	0.676
	Age	0.077	1.653	0.098
	BAI group (poor)	10.142	7.168	<0.001^∗^
	Time∗BAI group	−0.009	−4.293	<0.001^∗^

BDI	Intercept	−0.416	−0.203	0.839
	Time	−0.001	−0.786	0.432
	Age	0.108	2.484	0.013^∗^
	BDI group (poor)	11.258	8.551	<0.001^∗^
	Time∗BDI group	−0.009	−4.744	<0.001^∗^

ESS	Intercept	6.227	6.030	<0.001^∗^
	Time	0.0001	−0.699	0.485
	Age	−0.008	−0.350	0.726
	ESS group (poor)	6.481	7.469	<0.001^∗^
	Time∗ESS group	−0.001	−0.661	0.509

PSQI	Intercept	2.667	2.496	0.013^∗^
	Time	0.001	1.614	0.107
	Age	0.045	2.057	0.040^∗^
	PSQI group (poor)	5.026	7.039	<0.001^∗^
	Time∗PSQI group	−0.002	−1.939	0.052

## Data Availability

The data used to support the findings of this study are available from the corresponding author upon request.
